# Effects of Varying N, P, K, Mg, and Ca Concentrations on Nitrogen Transport in Xylem Sap of Rice Plants

**DOI:** 10.3390/plants14081154

**Published:** 2025-04-08

**Authors:** Shohei Watado, Kyoko Higuchi, Akihiro Saito, Takuji Ohyama

**Affiliations:** 1 Laboratory of Biochemistry in Plant Productivity, Department of Agricultural Chemistry, Tokyo University of Agriculture, Setagaya-ku, Tokyo 156-8502, Japankhiguchi@nodai.ac.jp (K.H.); a3saito@nodai.ac.jp (A.S.); 2 Department of Agriculture, Niigata University, Niigata 950-2102, Japan

**Keywords:** asparagine, Ca, glutamine, K, Mg, N, nitrate, P, rice, xylem sap

## Abstract

The nutrients absorbed in the plant roots are transported to the shoots through the xylem. The effects of concentrations of N, P, K, Mg, and Ca in a culture solution on N transport forms have not been fully elucidated. In this study, rice plants were grown with five concentrations of N, P, K, Mg, and Ca for three days, and the concentrations of major N compounds in the xylem sap were determined. In the control plants, nitrate, glutamine, and asparagine were the principal N compounds. The concentrations of nitrate, glutamine, and asparagine decreased consistently with a decrease in the N concentration in the culture solution. Different P concentrations did not affect the N components. With lower K concentrations, only the nitrate concentration decreased. While the glutamine and asparagine concentrations decreased with a decrease in the Mg concentration. The Ca concentration did not affect the N concentration, except for Ca deprivation. The citrate and malate concentrations markedly increased when the plants grew with an N-free solution due to regulating the cation-anion balance. The results of this study indicate that changes in the concentrations of N, K, Mg, and Ca affected the concentrations of N transport forms, especially nitrate, glutamine, and asparagine.

## 1. Introduction

Rice (*Oryza sativa* L.) is a staple food for more than half of the world’s population, providing over 20% of the calories consumed worldwide, and about 90% of rice is produced in Asia [1,2]. Although upland rice is cultivated, most rice is produced in submerged paddy fields. Rice production must double by 2050 to meet the demand of an increasing world population; however, the current rice production bottleneck is the ceiling on yields [3].

Nitrogen (N) is the most crucial nutrient in rice production, affecting plant growth and seed yield. Paddy rice is grown in hypoxic or anaerobic submerged fields, and the primary form of inorganic N in the soil is ammonium. Ammonium uptake into the root cell is known to be facilitated by ammonium transporters located in the plasma membrane. In rice, OsAMT1;1, OsAMT1;2, and OsAMT1;3 play crucial roles in root ammonium uptake [4]. The expressions of OsAMT1;1 and OsAMT1;2 are up-regulated in response to high ammonium concentrations, whereas OsAMT1;3 is expressed under N deprivation, suggesting that its function in rice is to adapt to low-ammonium environments [5].

As a high concentration of NH_4_^+^ in cells is toxic [6], absorbed NH_4_^+^ needs to be rapidly assimilated into organic compounds. As shown in early tracer studies using ^15^NH_4_^+^ [7,8], the NH_4_^+^ absorbed through rice roots is assimilated initially into the amide-N of glutamine (Gln) coupled with glutamate (Glu) by the enzyme glutamine synthetase (GS). The amide-N of Gln is transferred to 2-oxo-glutarate to produce two Glu molecules by glutamate synthase (GOGAT). Gln and Glu are the precursors of various N components in plants, such as amino acids, amides, and nucleic acids [9]. Asparagine (Asn) is synthesized with the amide-N of Gln or NH_4_^+^ and aspartate (Asp) by the enzyme asparagine synthetase (AS).

Rice plants grown under submerged conditions can absorb NO_3_^−^ when NO_3_^−^ is applied as a fertilizer [10,11]. The rice plants grown under upland conditions absorb the NO_3_^−^ supplied by soil. Alternatively, NO_3_^−^ can be provided under flooded conditions, as rice plants can transfer O_2_ from their shoots to their roots through the aerenchyma and release it to the rhizosphere via special aerated tissue cells; thus, nitrification may occur in the root surface [12,13,14]. NO_3_^−^ uptake is known to be mediated by nitrate transporters localized in the plasma membrane of the root epidermal cells or cortical cells. *OsNRT2.1*, *OsNRT2.2*, and *OsNRT2.3a*, belonging to the *OsNRT2* family, are transcriptionally up-regulated by NO_3_^−^ supply and require a partner protein, OsNAR2.1 for nitrate uptake [15,16,17,18]. Some part of the absorbed NO_3_^−^ is reduced in the roots by nitrate reductase (NR) and nitrite reductase (NiR) to ammonium, and the other NO_3_^−^ is transported to the shoot via the xylem and reduced and assimilated in the leaves.

Concerning the principal transporters of other nutrients in rice, Jia et al. [19] reported that the phosphate transporter OsPht1;8 is involved in phosphate homeostasis in rice. In addition, the rice OsHAK16 mediates K uptake and translocation from root to shoot and may play crucial roles in maintaining K homeostasis and salt tolerance in rice shoots [20]. There are nine homologs of magnesium transporters in rice [21], and OsMGT1 is a transporter for Mg uptake in rice roots [21,22]. A spatial expression analysis revealed that *OsMGT1* was expressed in all root cells of the root tips but was highly expressed in the pericycle of mature zone roots [22]. Ca is indispensable for the growth and development of plants and animals, and a profile-based search program identified 44 Ca transporter genes [23].

The xylem and phloem are two major long-distance transport pathways between plant organs [9]. Water and nutrients absorbed in the roots are transported upward via the xylem vessels to the shoot by the driving forces of transpiration and root pressure. On the other hand, photoassimilates, amino acids, and mineral nutrients in the leaves are translocated via phloem to the roots and growing parts of the shoot as apical parts, developing leaves, and reproductive parts, such as flowers and fruits [9]. Yoneyama et al. [24,25,26] studied the N absorption and transport in rice plants using ^15^N as a tracer and revealed that the appreciable amount of ^15^N-labeled N incorporated into mature leaves was re-translocated to the youngest leaves [24]. Amino acids are the principal form of N re-translocated from the matured leaves to the youngest leaves, and Arg, Lys, Pro, and Val might be derived from re-translocated nitrogen, although Glu, Asp, and Ala might depend on the newly absorbed N from roots [25,26]. Concerning the long-distance N transport forms of rice plants, Fukumorita and Chino [27] reported that Gln (33% of total amino acid on a molar basis) and Asn (13%), Ser (9%), Glu (7%), and Asp (4%) were the principal N transport forms in the xylem sap of rice plants supplied with ammonium nitrate in a culture solution. The authors also reported that asparagine (17–33% on a molar basis), glutamate (6–14%), serine (10–13%), glutamine (7–15%), threonine (5–6%), and valine (6–7%) were dominant in the phloem sap collected from rice leaf sheath through severed stylets of brown planthoppers [27]. On the other hand, Hayashi et al. [28] reported that NO_3_^−^ was the main transport form in the xylem of rice plants supplied with NO_3_^−^, and more than 90% of N translocated was amino acids in the pure phloem sap of rice plants. Kiyomiya et al. [29] visualized the real-time translocation of ^13^N in rice plants using a positron-emitting tracer imaging system, and they identified an accumulation of ^13^N signals detected within 2 min at the basal node of the shoots, which was named a “discrimination center”. 

In this study, rice plants were hydroponically grown in a culture solution and treated with five concentrations, ×0, ×0.2, ×0.5, ×1, and ×5, of N, P, K, Mg, or Ca of the control solution for three days, and the xylem sap bled from the cut stump was collected for an hour. The concentrations of amides, amino acids, organic acids, inorganic cations, and inorganic anions in the xylem sap were analyzed. The goal is to understand how the concentrations of N transport forms are affected by deficient, adequate, or surplus concentrations of major nutrient elements in the culture solution. In addition, how the cation and anion balance in xylem sap was influenced and balanced under these treatments. Finally, we discuss the applicability of xylem sap analysis on the diagnosis of nutrient status in rice plants. To the best of our knowledge, this is the first report to investigate the effects of varying major nutrient elements on N transport forms in the xylem sap in rice plants.

## 2. Results

### 2.1. Xylem Sap Exudation Rates and Transpiration Rates with Various Treatments

**Figure 1:**
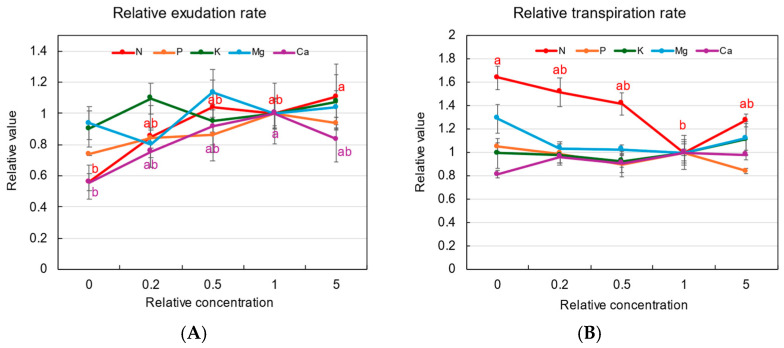
Relative xylem sap exudation rate (**A**) and relative transpiration rate (**B**) values of rice plants with 3 days of N, P, K, Mg, or Ca treatments. The value for each treatment was divided by that grown in the standard solution. The different letters of the same color with the symbols near the plot indicate significant differences among the different concentrations in each treatment based on Tukey’s test (*p* < 0.05). Letters are omitted in cases in which there was no significant difference among the concentrations. Average ± standard error. *N* = 4. The values on the *Y*-axis are the ratios (no unit).

Figure 1A shows the relative exudation rates of the xylem sap in each treatment, where the exudation rates (mL/h/plant) of plants grown with different concentrations are divided by the exudation rate of the control plants (×1). After 3 days of treatments, the growth of plants was not significantly different. However, the relative xylem sap exudation rates at ×0 concentration in the N and Ca treatments were lower than 0.6 compared with the control at ×1 concentration. The relative exudation rates in the N, K, and Mg treatments were not significantly different among concentrations in the culture solution.

Figure 1B shows the relative transpiration rates from the second to the third treatment days. In the N treatment, the relative transpiration rates were higher at 1.6 times under ×0 N concentrations. The relative transpiration rates with P, K, Mg, and Ca were not significantly different among the various concentrations.

### 2.2. Concentrations of Treated Elements in Xylem Sap and Their Absorption Rates from the Culture Solution

**Figure 2:**
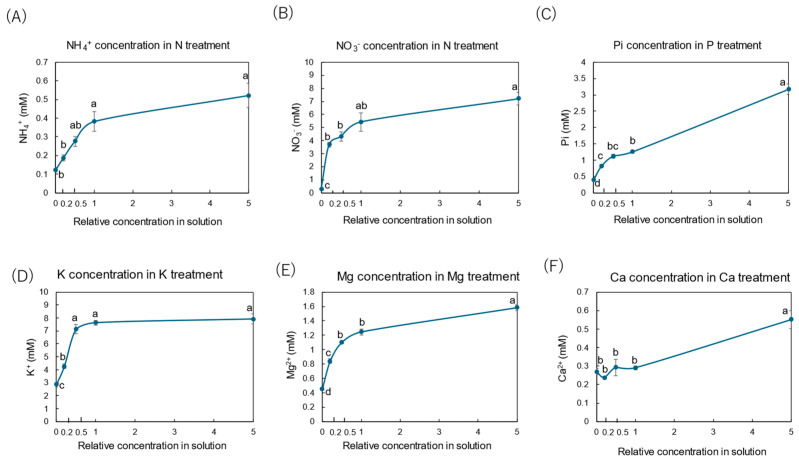
Concentrations of inorganic N, P, K, Mg, and Ca in the xylem sap collected after 3 days of each treatment. Average ± standard error. The relative concentrations in the solution on the *x*-axis indicate the ratio of each concentration to that of the standard concentration. The different letters near the plots indicate significant differences among the concentrations of each treatment based on Tukey’s test (*p* < 0.05). Average ± standard error. *N* = 4.

Figure 2 shows the xylem concentrations of NH_4_^+^ (Figure 2A) and NO_3_^−^ (Figure 2B) in the N treatment, inorganic P (Pi) (Figure 2C) in the P treatment, K^+^ (Figure 2D) in the K treatment, Mg^2+^ (Figure 2E) in the Mg treatment, and Ca^2+^ (Figure 2F) in the Ca treatment. In the N treatment, the concentration of NO_3_^−^ in the xylem was 5.5 mM at the control concentration (×1 N), much higher than the NH_4_^+^ concentration of 0.4 mM at ×1 N. The changes in the NH_4_^+^ and NO_3_^−^ concentrations in the xylem sap with the N treatment showed similar hyperbolic patterns against the N concentrations in the culture solution, such as in the Michaelis–Menten kinetics applied to determine the enzyme and substrate reaction rates [30]. When the concentration of N in the culture solution decreased, the NH_4_^+^ and NO_3_^−^ concentrations in the xylem sap decreased. The Pi concentration in the xylem sap in the control (×1 P) treatment was 1.2 mM and decreased when the Pi concentration in the culture solution decreased, showing a hyperbolic curve. When a high concentration (×5 P) of P was applied, the concentration of Pi in the xylem sap increased significantly to 3 mM. The K^+^ concentration in the xylem sap for the control (×1 K) concentration was 7.5 mM and decreased when the K concentration decreased to ×0.2 K and ×0 K. At the higher concentration of K (×5 K) in the culture solution, the concentration of K did not differ from the control (×1 K), unlike those in the N and P treatments. The xylem sap Mg^2+^ concentration in the control (×1 Mg) concentration was 1.2 mM and decreased when the Mg^2+^ concentration in the culture solution decreased, showing a hyperbolic curve. When a higher concentration of Mg (×5 Mg) was applied, the Mg^2+^ concentration in the xylem sap was about 1.6 mM and was significantly higher than that for the control (×1 Mg). The Ca^2+^ concentration in the control (×1 Ca) was about 0.3 mM and was constant at ×0 Ca, ×0.2 Ca, ×0.5 Ca, and ×1 Ca. When a higher-concentration Ca solution was applied (×5 Ca), the Ca^2+^ concentration in the xylem sap increased significantly to 0.6 mM. The Ca^2+^ concentration appeared to increase linearly, not hyperbolically, with the relative concentration of Ca in the solution.

**Figure 3:**
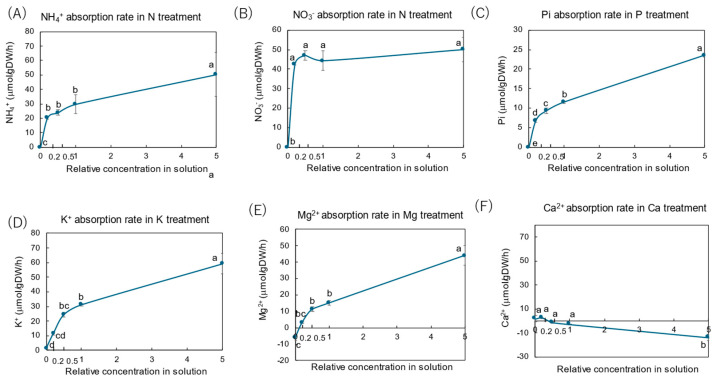
Absorption rates of inorganic N, P, K, Mg, and Ca in the culture solution from days 2 to 3. Average ± standard error. The different letters near the plots indicate significant differences among the concentrations of each treatment based on Tukey’s test (*p* < 0.05). Average ± standard error. *N* = 4.

Figure 3 shows the absorption rates of NH_4_^+^ and NO_3_^−^ in the N treatment, those of Pi in the P treatment, those of K^+^ in the K treatment, those of Mg^2+^ in the Mg treatment, and those of Ca^2+^ in the Ca treatment. The absorption rates of the elements were calculated from the balance of each nutrient content in a bottle before and after cultivation from the second day to the third day of the treatments. The absorption rates were determined based on the DW in grams of roots per hour. In the N treatment (Figure 3A,B), the absorption rates of NH_4_^+^ and NO_3_^−^ in the control (×1 N) solution were 30 and 43 μmol/g DW/h, respectively. At lower concentrations of N (×0.5 N, ×0.2 N), the absorption rates of NH_4_^+^ and NO_3_^−^ were not significantly changed, but both were negligible for the ×0 N solution. The absorption rate of NH_4_^+^ at the ×5 N concentration was higher than that for the control ×1 N, but that of NO_3_^−^ was constant from the ×0.2 N to the ×5 N solution. In the P treatment (Figure 3C), the absorption rate of Pi in the control (×1 P) solution was 12 μmol/g DW/h. At lower concentrations of P (×0.5 P, ×0.2 P), the Pi absorption rate decreased significantly, but it increased to 23 μmol/g DW/h in higher concentrations (×5 P). In the K treatment (Figure 3D), the K^+^ absorption rate in the control (×1 K) solution was 31 μmol/g DW/h, showing a similar pattern against concentration changes in the culture solution as shown by NH_4_ (Figure 3A) and Pi (Figure 3C). In the Mg treatment (Figure 3E), the Mg^2+^ absorption rate in the control (×1 Mg) solution was 12 μmol/g DW/h and decreased with lower Mg concentrations in the culture solution, but it increased to 40 μmol/g DW/h in the ×5 Mg concentration. In the Ca treatment (Figure 3F), the Ca^2+^ absorption rate decreased and showed negative values. This result might be an experimental error due to a low absorption rate or caused by an increase in Ca^2+^ efflux.

### 2.3. Concentration of Major N Compounds in Xylem Sap of Rice Plants Treated with Different Concentrations of Major Elements

**Figure 4:**
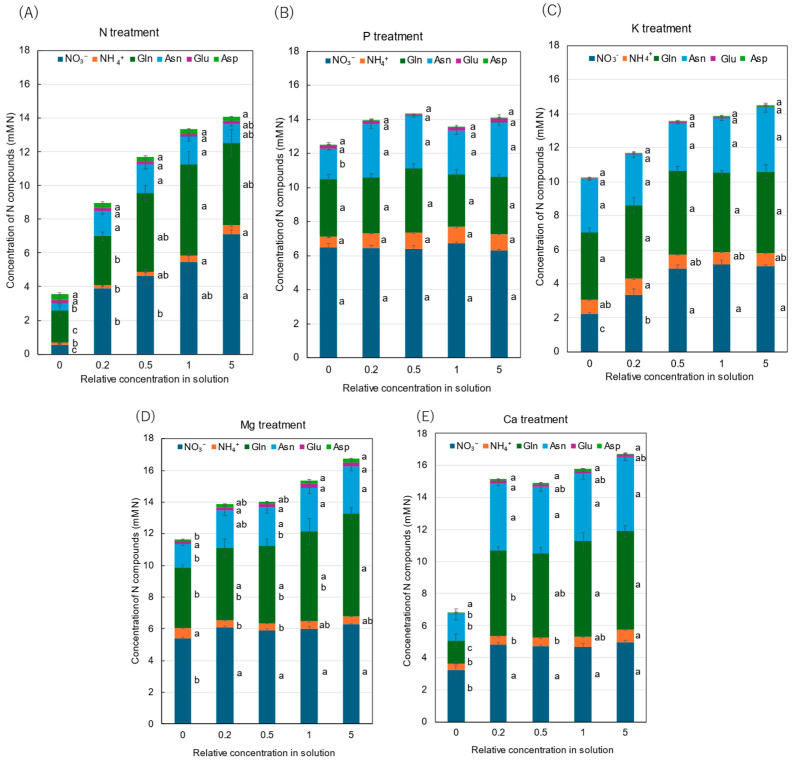
Concentrations of principal N compounds in the xylem sap of rice plants treated with different concentrations of N, P, K, Mg, and Ca treatments. Average ± standard error. The different letters beside the columns indicate significant differences in the concentrations of N compounds among the concentrations of each treatment based on Tukey’s test (*p* < 0.05). Average ± standard error. *N* = 4. Abbreviations: Gln: glutamine, Asn: asparagine, Glu: glutamate, and Asp: aspartate.

Figure 4 shows the concentrations of the major N compounds in the xylem sap collected from rice plants grown with various concentrations of N, P, K, Mg, and Ca for three days. The average N concentrations of NO_3_^−^, NH_4_^+^, Gln, Asn, Glu, and Asp in the control treatment were 5.42, 0.57, 4.76, 2.96, 0.12, and 0.11 mM N, respectively. NO_3_^−^, Gln and Asn were the principal N compounds in the xylem sap, and the percentage accounted for 39%, 34%, and 21% of the sum of the analyzed N compounds. The NH_4_^+^ concentration was relatively low (4.1%), and the concentrations of Glu (0.8%) and Asp (0.8%) were much lower.

After 3 days of N treatment (Figure 4A), the concentrations of NO_3_^−^, NH_4_^+^, Gln, and Asn in the xylem sap decreased with a decrease in the N concentration in the culture solution. At ×0 N concentration, the N concentrations of NO_3_^−^, NH_4_^+^, Gln, and Asn were 0.55, 0.12, 1.92, and 0.50 mM N, where the values were significantly lower than those in the control concentration (×1 N). On the other hand, the Glu and Asp concentrations did not change among concentration treatments.

In the P treatment (Figure 4B), the concentrations of NO_3_^−^, NH_4_^+^, Gln, Asn, Glu, and Asp were almost the same among all concentrations from ×0 P to ×5 P, except for the Asn concentration at ×0 P.

In the K treatment (Figure 4C), the concentrations of all compounds were almost the same among the ×0.5 K, ×1 K, and ×5 K concentrations. However, the concentration of NO_3_^−^ was significantly decreased by the ×0.2 K and ×0 K concentrations compared with the control (×1 K). The concentrations of NH_4_^+^, Gln, Asn, Glu, and Asp were not different among the K concentrations.

In the Mg treatment (Figure 4D), the concentrations of Asn and Gln decreased with a decrease in the Mg concentration in the culture solution. The concentrations of NO_3_^−^ and NH_4_^+^ were almost the same among the Mg concentrations in the culture solution, except for the NO_3_^−^ concentration at ×0 Mg.

Figure 4E shows the concentrations of various N forms in the xylem sap collected from rice plants grown with different concentrations of Ca. The concentrations of NO_3_^−^, NH_4_^+^, Gln, Asn, Glu, and Asp were almost the same from ×0.2 Ca to ×5 Ca; however, under Ca-deprivation conditions (×0 Ca), the concentrations of NO_3_^−^, Gln, and Asn were significantly lower than those with the control (×1 Ca) plants.

**Figure 5:**
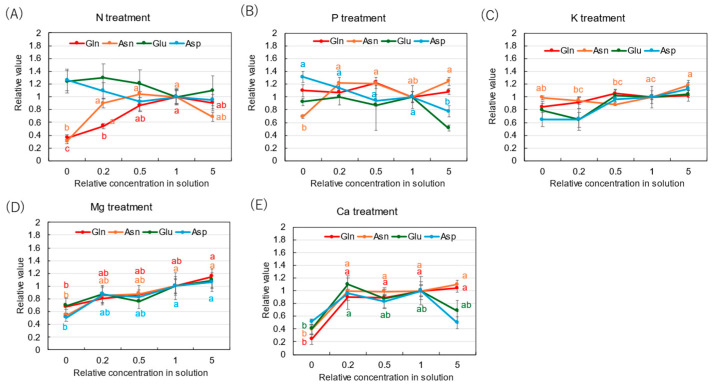
Relative concentrations of amino acids in xylem sap of rice plants after 3 days of cultivation of different concentrations of N, P, K, Mg, or Ca in the culture solution. The value for each treatment was divided by that for the standard solution. The different letters of the same color with the symbols near the plot indicate significant differences among the different concentrations in each treatment based on Tukey’s test (*p* < 0.05). Letters are omitted in cases in which there was no significant difference among the concentrations. *N* = 4. Average ± standard error. *N* = 4. The values on the *Y*-axis are the ratios (no unit). Abbreviations: Gln: glutamine, Asn: asparagine, Glu: glutamate, and Asp: aspartate.

Figure 5 shows the relative concentrations of amides and amino acids in the xylem sap, where the concentrations of each compound in ×0, ×0.2, ×0.5, and ×5 solutions were divided by the concentration for the control (×1). In the N treatment (Figure 5A), the relative concentrations of Gln and Asn decreased as the N concentration in the culture solution decreased. At ×0 N, the relative concentrations of Gln and Asn were very low, at 0.35 and 0.31, respectively. On the other hand, the relative concentrations of Glu and Asp did not decrease with ×0.2 N and ×0 N. When a high concentration of N was supplied (×5 N), the relative concentrations of Gln, Asn, Glu, and Asp were the same as those in ×1 N. The relative concentrations of amides and amino acids in the P treatment (Figure 5B) showed that the relative concentration of Asn at ×0 P was 0.69, which is notably lower compared with the control ×1 P. In the K treatment (Figure 5C), the relative concentrations of Gln, Asn, Glu, and Asp were constant in the ×0.5 K, ×1 K, and ×5 K concentrations. In the Mg treatment (Figure 5D), the relative concentrations of all compounds changed similarly and decreased with the lower concentrations in the culture solution. In the Mg deficiency conditions at ×0 Mg, the relative concentrations of Asp (0.51) and Asn (0.54) were low. In the Ca treatment (Figure 5E), the relative concentrations of Gln and Asn were similar among ×1 Ca, ×0.5 Ca, and ×0.2 Ca. However, those decreased at ×0 Ca, where the relative concentrations of Gln (0.24), Asn (0.39), Glu (0.40), and Asp (0.51) were low.

### 2.4. Organic Acid Concentrations in Xylem Sap with Various Concentrations of Major Elements

**Figure 6:**
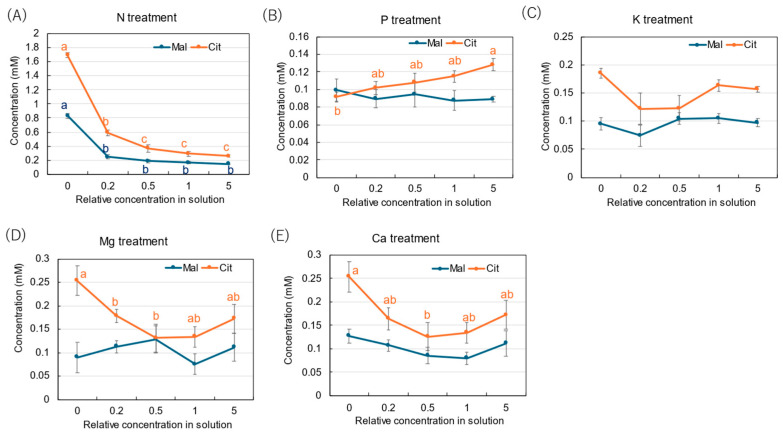
Concentrations of malate and citrate in the xylem sap of rice plants treated with different concentrations of N, P, K, Mg, and Ca treatments for 3 days. The different letters of the same color with the symbols near the plot indicate significant differences among the different concentrations in each treatment based on Tukey’s test (*p* < 0.05). Letters are omitted in cases in which there was no significant difference among the concentrations. Average ± standard error. *N* = 4.

The average concentrations of citrate (Cit) and malate (Mal) were 0.17 mM and 0.10 mM in the xylem sap collected from the control ×1 concentration. In the N treatment (Figure 6A), the Cit and Mal concentrations increased to 1.69 mM and 0.83 mM with ×0 N in the culture solution. On the other hand, the P treatment (Figure 6B) did not show significant changes, except with ×0 P. In the K treatment (Figure 6C), the concentrations of Cit and Mal did not change significantly among the K concentrations in the culture solution. In the Mg (Figure 6D) and Ca (Figure 6E) treatments, only the Cit concentration increased with low Mg and Ca concentrations in the culture solution, especially at the ×0 Mg (0.25 mM) and ×0 Ca (0.25 mM) concentrations.

### 2.5. Comparison of the Equivalent Concentrations of Anions and Cations in the Xylem Sap of Plants Cultivated with Various Concentrations of N, P, K, Mg, and Ca

In the control ×1 concentrations, the average equivalent concentrations of NO_3_^−^, Pi, SO_4_^2−^, Cl^−^, Cit, and Mal in the xylem sap with all treatments were 5.24, 1.57, 1.75, 0.22, 0.36, and 0.24 mEq, respectively, which accounted for 56%, 17%, 19%, 2.3%, 3.9%, and 2.5% of the total anions. The average equivalent concentrations of NH_4_^+^, K^+^, Na^+^, Mg^2+^, and Ca^2+^ were 0.64, 8.37, 0.04, 2.44, and 0.63 mEq, which accounted for 5%, 69%, 0.3%, 20%, and 5% of the total cations. The K^+^ concentration was very high, and it was the primary cation in the xylem sap, followed by Mg^2+^, NH_4_^+^, and Ca^2+^. The Na^+^ concentration was very low, possibly because only a small amount of Na^+^ was supplied from Fe(III)EDTA Na.

**Figure 7:**
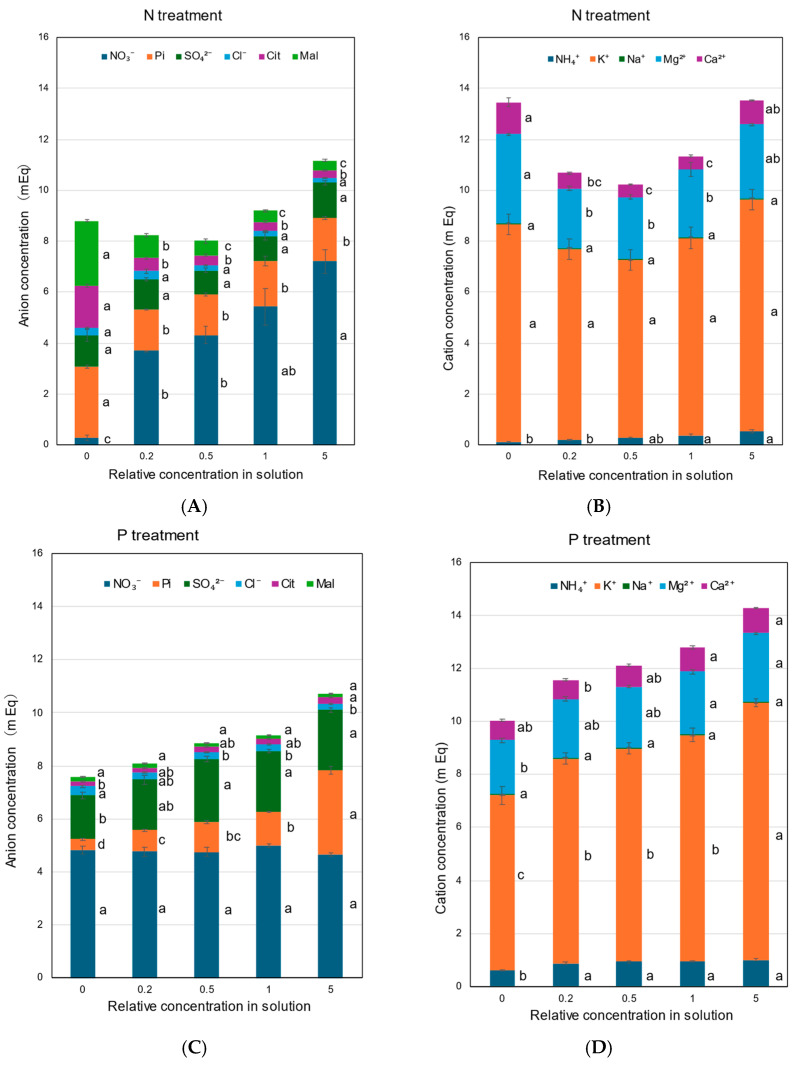

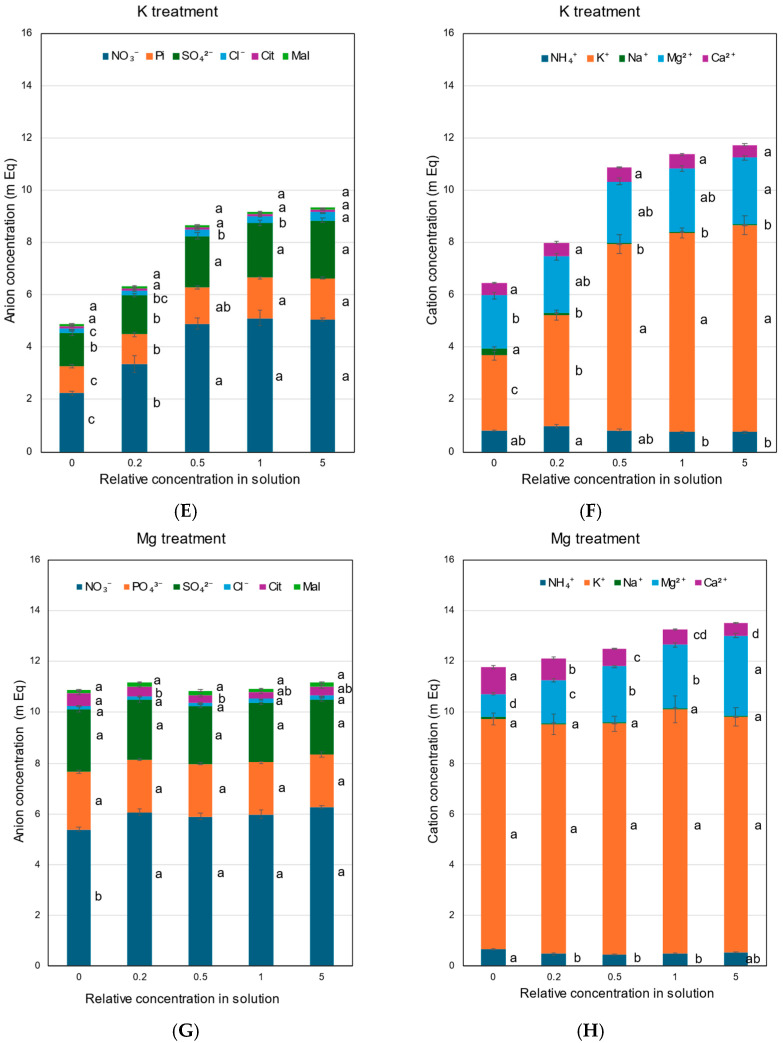

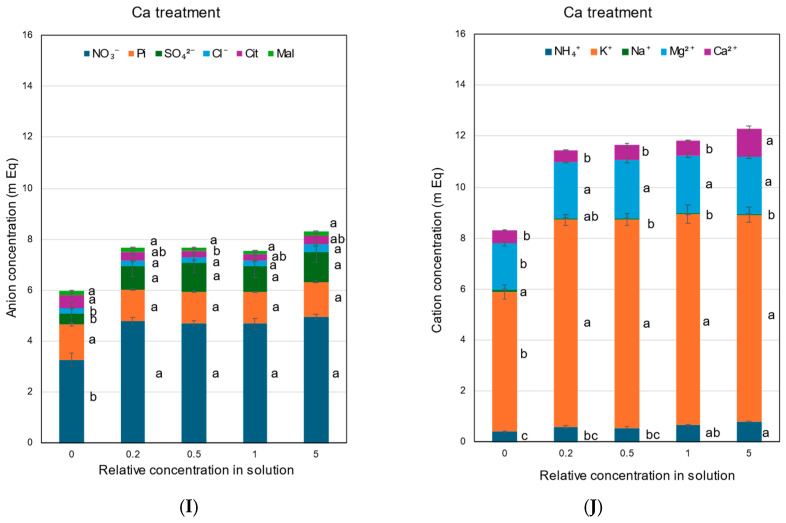
Concentrations of the equivalent anions and cations in the xylem sap of rice plants treated with different concentrations of N, P, K, Mg, and Ca for 3 days. Average ± standard error. The different letters beside the columns indicate significant differences in the m Eq concentration among the concentrations of each treatment based on Tukey’s test (*p* < 0.05). Average ± standard error. *N* = 4. (**A**) anions in the N treatment, (**B**) cations in the N treatment, (**C**) anions in the P treatment, (**D**) cations in the P treatment, (**E**) anions in the K treatment, (**F**) cations in the K treatment, (**G**) anions in the Mg treatment, (**H**) cations in the Mg treatment, (**I**) anions in the Ca treatment, and (**J**) cations in the Ca treatment.

The concentrations of anions, NO_3_^−^, Pi, SO_4_^2−^, Cl^−^, Cit, and Mal, in the xylem sap collected with the N treatment are shown in Figure 7A. In the N treatment, the concentration of NO_3_^−^ decreased as the solution N concentration decreased. The concentration of Pi did not change among the ×0.2 N, ×0.5 N, ×1 N, and ×5 N concentrations, but it increased at the ×0 N concentration. The concentrations of SO_4_^2−^ and Cl^−^ in the xylem sap were not significantly changed. The concentrations of Cit and Mal markedly increased as the N concentration decreased in the culture solution. At ×0 N, the concentrations of Mal and Cit were significantly high, at 2.8 and 1.7 mEq, respectively. The concentrations of inorganic cations, NH_4_^+^, K^+^, Na^+^, Mg^2+^, and Ca^2+^, in the N treatment are shown in Figure 7B. The K^+^ concentrations were always highest among the N concentrations in the culture solution and did not change significantly. The NH_4_^+^ concentrations in the xylem sap were relatively low but steadily increased with higher concentrations of N in the culture solution. At the ×0 N concentration, the Mg^2+^ and Ca^2+^ concentrations in the xylem sap were significantly higher than those in the control at ×1 N.

In the P treatment (Figure 7C), the Pi concentrations in the xylem sap consistently decreased with a decrease in the P concentration from ×1 P (1.25 mEq) to ×0.5 P (1.12 mEq), ×0.2 P (0.81 mEq), and ×0 P (0.40 mEq). The concentration of Pi in the ×5 P concentration was 3.17 mEq and significantly higher than that in the control (×1 P). The NO_3_^−^ concentrations were relatively constant among the P concentrations in the culture solution. The concentration of SO_4_^2−^ at ×0 P was lower than that at the other concentrations, but the Cl^−^ concentration at ×0 P was higher than that with the control (×1 P). The higher ×5 P concentration did not affect the concentrations of SO_4_^2−^ and Cl^−^ compared with ×1 P. The P treatment did not affect the Cit and Mal concentrations. As shown in Figure 7D, the K^+^ concentrations gradually decreased with lower P concentrations in the culture solution. At ×0 P, the concentration of K^+^ was significantly lower (6.62 mEq) than that in ×1 P (8.55 mEq). The K^+^ concentration at ×5 P was 9.73 mEq and higher than that in the control (×1 P). At ×0 P, the concentrations of NH_4_^+^ and Mg^2+^ were significantly lower than those for the control (×1 P), but the mEq concentrations of Na^+^ and Ca^2+^ were not.

In the K treatment, the NO_3_^−^ concentration decreased at ×0.2 K (3.4 mEq) and ×0 K (2.2 mEq) compared with the control ×1 (5.1 mEq) (Figure 7E). The concentrations of Pi, SO_4_^2−^, Cl^−^, Cit, and Mal in the xylem sap and those of inorganic cations, NH_4_^+^, K^+^, Na^+^, Mg^2+^, and Ca^2^ (Figure 7F), were almost constant among ×0.5 K, ×1 K, and ×5 K. However, the concentrations of anions and cations decreased at ×0.2 K, and the lowest concentrations at ×0 K.

Figure 7G,H show the concentrations of anions and cations in the xylem sap with the Mg treatment. The concentrations of anions were constant among the Mg concentrations in the culture solution, except for NO_3_^−^ at ×0 Mg. The Mg^2+^ concentrations in the xylem sap changed with the Mg concentrations in the culture solution, lowest at ×0 Mg (0.92 mEq) and highest at ×5 Mg (3.17 mEq). On the other hand, the Ca^2+^ concentrations increased with a decrease in the Mg concentrations in ×0.2 Mg (0.87 mEq) and ×0 Mg (1.07 mEq), compared with the control ×1 Mg (0.62 mEq). The concentrations of K^+^ in the xylem sap did not change among the Mg concentrations in the culture solution.

The concentrations of anions (Figure 7I) and cations (Figure 7J) under Ca treatments were constant among ×0.2 Ca, ×0.5 Ca, ×1 Ca, and ×5 Ca, except for the Ca^2+^ concentrations. The Ca^2+^ concentrations were higher in ×5 Ca than in ×0 Ca, ×0.2 Ca, and ×0.5 Ca. At ×0 Ca, the concentrations of NO_3_^−^ were significantly lower than those in the control ×1 Ca. At ×0 Ca, NH_4_^+^, K^+^, and Mg^2+^ concentrations were markedly lower than the control ×1 Ca. The cation concentrations were relatively constant at ×0.2 Ca, ×0.5 Ca, ×1 Ca, and ×5 Ca.

**Figure 8:**
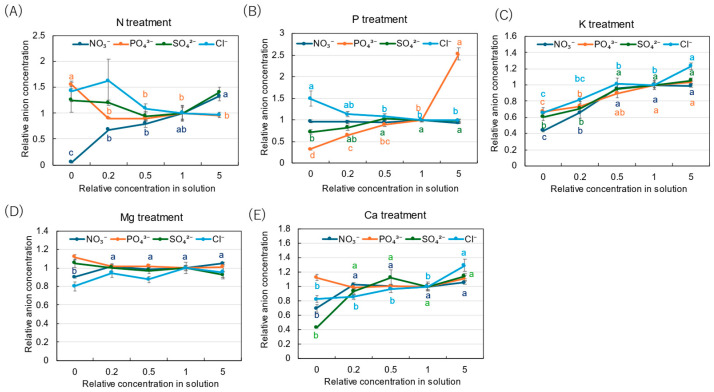
Relative concentrations of inorganic anions in the xylem sap of rice plants after 3-day cultivation of different concentrations of N, P, K, Mg, or Ca in the culture solution. The different letters of the same color with the symbols near the plot indicate significant differences among the different concentrations in each treatment based on Tukey’s test (*p* < 0.05). Letters are omitted in cases in which there was no significant difference among the concentrations. The value for each treatment was divided by that for the standard solution. Average ± standard error. *N* = 4. The values on the *Y*-axis are the ratios (no unit).

The relative concentrations of inorganic anions are shown in Figure 8 to clarify the concentration effects of N, P, K, Mg, and Ca in the culture solution. The relative concentrations of NO_3_^−^ decreased with a decrease in the N concentration in the solution, and the relative concentration was only 0.05 at 0 N (Figure 8A). On the other hand, the relative concentrations of Pi tended to increase with a decrease in the N concentration, especially at ×0 N. In the P treatment (Figure 8B), the relative concentrations of Pi decreased with a decrease in the P concentration in the culture solution, and the value was the lowest at ×0 P (0.32). The relative concentration of Pi was significantly high, with a value of 2.53 at ×5 P. In the K treatment (Figure 8C), the relative concentrations of all anions decreased with a decrease in the K concentration, while they were relatively constant at ×5 P. In the Mg treatment (Figure 8D), the relative concentrations of all anions were not affected by the Mg concentration in the culture solution. In the Ca treatment (Figure 8E), the relative concentrations of all anions among the ×0.2 Ca, ×0.5 Ca, ×1 Ca, and ×5 Ca concentrations were constant. However, the relative concentrations of SO_4_^2−^ (0.43), NO_3_^−^ (0.70), and Cl^−^ (0.83) decreased at ×0 Ca compared with the control (×1 Ca).

**Figure 9:**
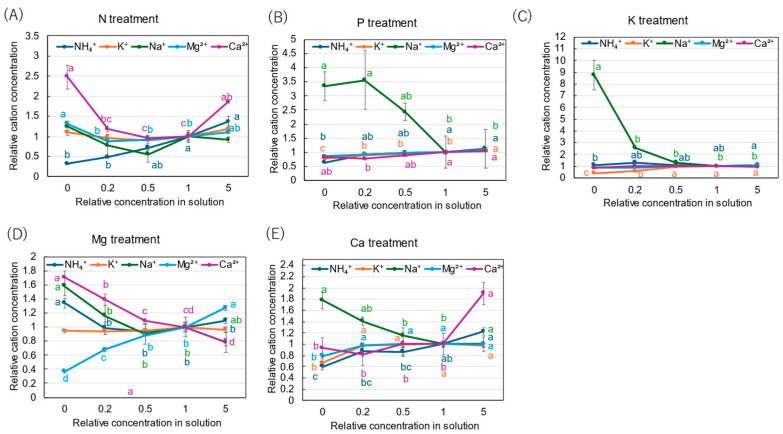
Relative concentrations of inorganic cations in the xylem sap of rice plants after 3-day cultivation of different concentrations of N, P, K, Mg, or Ca in the culture solution. The value for each treatment was divided by that for the standard solution. The different letters of the same color with the symbols near the plot indicate significant differences among the different concentrations in each treatment based on Tukey’s test (*p* < 0.05). Letters are omitted in cases in which there was no significant difference among the concentrations. Average ± standard error. *N* = 4. The values on the *Y*-axis are the ratios (no unit).

The relative concentrations of inorganic cations in the xylem sap with N, P, K, Mg, and Ca are shown in Figure 9. In the N treatment (Figure 9A), the relative concentration of NH_4_^+^ decreased with a decrease in the N concentration in the culture solution. It was the lowest at ×0 N (0.32). On the other hand, the relative concentration of Ca^2+^ was higher at ×0 N (2.48) and ×5 N (1.85) compared with the control (×1 N). In the P treatment (Figure 9B), the relative concentrations of NH_4_^+^, K^+^, Mg^2+^, and Ca^2+^ were relatively constant among the P concentrations in the culture solution, but that of Na^+^ was significantly high, with 3.56 at ×0.2 P and 3.35 at ×0 P. In the K treatment (Figure 9C), the relative concentration of K^+^ decreased with a decrease in the K concentration in the culture solution, and the values were 0.56 at ×0.2 K and 0.38 at ×0 K. The relative concentrations of NH_4_^+^, Mg^2+^, and Ca^2+^ were relatively constant among the K concentrations in the culture solution, but that of Na^+^ was significantly high, with a value of 8.76 at ×0 P. In the Mg treatment (Figure 9D), the relative concentrations of Mg^2+^ decreased with a decrease in the Mg concentration in the culture solution, showing the lowest value of 0.37 at ×0 Mg. On the other hand, the relative concentrations of Ca^2+^ (1.70), Na^+^ (1.58), and NH_4_^+^ (1.34) increased at ×0 Mg. The relative concentrations of K^+^ were constant among the Mg concentrations in the culture solution. In the Ca treatment (Figure 9E), the relative concentrations of Ca^2+^ were relatively constant among ×0 Ca, ×0.2 Ca, ×0.5 Ca, and ×1 Ca but significantly increased to 1.90 at ×5 Ca. At ×0 Ca, the relative concentrations of NH_4_^+^ (0.60), K^+^ (0.66), and Mg^2+^ (0.79) decreased, while the relative concentration of Na^+^ increased with a decrease in the Ca concentration, resulting in a value of 1.78 at ×0 Ca.

## 3. Discussion

### 3.1. Effects of Major Nutrient Concentrations on Xylem Sap Exudation Rates and Transpiration

The upward transport of xylem sap from the roots to the shoots is driven by root pressure and transpiration. Several methods have been proposed to collect the xylem sap from various plants [31,32,33]. Among them, xylem sap collection from the cut end of the basal part of the shoot is the simplest method, and this technique can be readily applied to field experiments [34,35]. The motive force for the xylem sap exudation from a cut stump depends only on the root pressure because there is no transpiration after decapitation. Morita and Abe [34] investigated the xylem sap flow rate (mL/h/plant) to evaluate the physiological activity of whole root systems of rice plants grown in fields. They reported that the root bleeding rate was relatively constant during the day and night but tended to be higher in the morning compared with the afternoon [34,36]. Therefore, they collected xylem sap for 1 h in the morning [37]. During the growing season, the bleeding rate gradually increased to the maximum around the heading stage and rapidly decreased thereafter [34,37]. A positive relationship between rice yield and the bleeding rate at the heading stage has been reported in several studies [34,38,39]. Ju et al. [40] reported that the exudation rate was correlated positively with the number of productive stems in two rice varieties.

In this study, 3 days of depletion of N and Ca in the culture solution significantly reduced the xylem sap exudation rate (Figure 1A). However, similar trends were not observed with the depletion of K, P, and Mg. N is essential to support the synthesis of proteins such as transporters and enzymes; therefore, a decrease in the N concentration might decrease the xylem sap exudation rate only after 3 days of N depletion. On the contrary, the transpiration rate increased with a decrease in the N concentration in the culture solution (Figure 1B). The physiological mechanism promoting the transpiration rate under N deficiency is unknown; however, it may be related to an adaptation to acquire N by promoting the transpiration rate under N deficiency. Qi et al. [41] reported that when rice plants were grown in a control treatment (1.6 mM NO_3_^−^ plus 1.6 mM NH_4_^+^) and with ×1/2 and ×1/4 concentrations of N for 21 days, the transpiration rates were higher in the control plants than in the ×1/2 and ×1/4 treatments. This discrepancy between ours and Qi et al. may be due to the long duration of the N treatment in the latter case, in which the dry weight of the plant’s shoots and roots decreased in the ×1/2 and ×1/4 treatments compared with the control treatment (×1).

Both the relative xylem sap exudation rates and the relative transpiration rates were not significantly affected by the P, K, and Mg concentrations. These results might be attributed to the physiological status of the plants, meaning that the existing storage of P, K, and Mg might be sufficient to support physiological processes during a 3-day decrease in supply. In soybean plants cultivated in P-deficient conditions, relatively high concentrations of Pi were found to be stored in all the organs of nodulated soybean plants [42]. According to the results of K depletion treatments, K^+^ is generally the most abundant cation, and storage of K^+^ in the vacuoles of root cells or recycled K^+^ via phloem may compensate for a decrease in K^+^ absorption from the culture solution.

Ca deficiency caused a decrease in the relative exudation rate. Ca plays an important role in maintaining cell membrane integrity, and it is also important as a second messenger in the signal transduction between environmental factors and plant responses [43]. In addition, it was reported that in the absence of an exogenous Ca supply, root growth ceases within a few hours [43,44]. These essential roles of Ca might cause a decrease in the xylem sap exudation rate in Ca-deficient plants.

### 3.2. Effects of Major Nutrient Concentrations on Concentrations of Treated Elements in Xylem Sap and Absorption Rates

The patterns of the concentrations of NH_4_^+^, NO_3_^−^, Pi, and K^+^ in the xylem sap versus the concentration of treated elements (Figure 2) resembled the curves of their absorption rates (Figure 3), which significantly decreased following 3-day treatments with the lowered concentrations of each element compared with the control. The absorption rates of NH_4_^+^, NO_3_^−^, Pi, and K^+^ in the roots are mediated by specific transporters, which are likely to exhibit Michaelis–Menten-like kinetics [30]. The concentration changes in the xylem sap of plants grown with changing N, P, and K concentrations in the culture solution indicate that xylem sap concentration may be a good indicator of the absorption of these elements. Measuring the nutrient absorption rate in the field is very difficult; however, analyses of xylem sap collected from cut basal shoots are relatively easy to carry out; therefore, the analysis of xylem sap can be applied to the nutritional diagnosis of rice plants.

The patterns of the absorption rate of Mg^2+^ and the concentrations of Mg^2+^ in the xylem sap versus the Mg concentration in the culture solution showed a hyperbolic curve, such as in the N, P, and K treatments. Thus, Mg^2+^ absorption might be mediated through Mg^2+^ transporters in the rice roots. However, the concentration of Ca^2+^ in the xylem sap did not respond to lower concentrations in the culture solution and linearly increased at the high Ca concentration. The absorption rates of Ca^2+^ decreased to negative values as the Ca concentration of the solution increased. This result might be attributed to experimental errors because the Ca absorption rate was low and the Ca concentration in the culture solution was high. It was reported that the absorption and distribution of Ca in rice is very low, and almost 1/10 of dicotyledon plants such as tomatoes and cucumbers [45].

### 3.3. Effects of Major Nutrient Concentrations on Concentrations of N Compounds in Xylem Sap

The concentrations of NO_3_^−^ in the xylem sap responded positively to the N concentration in the culture solution with N treatment, and the concentration was much lower with the N-depleted conditions (×0 N) compared with other concentrations (Figure 4A). This result suggests that the turnover rate of storage NO_3_^−^ in the rice roots might be rapid and almost depleted after 3 days of ×0 N cultivation. The concentrations of NO_3_^−^ decreased with decreasing K concentrations in the culture solution (Figure 4C). This trend might be due to the control mechanism to equalize the electrical balance with cations and anions in the xylem sap. The decreasing K absorption might induce either the decrease in NO_3_^−^ absorption or the loading of NO_3_^−^ into xylem vessels, or both, although the physiological mechanism is unknown. The NO_3_^−^ concentration decreased under the Mg- and Ca-depleted conditions (Figure 4D,E), although there was no effect by the other concentrations of Mg and Ca. Mg^2+^ is essential for binding ATP to enzymes such as GS; therefore, Mg deficiency might affect the initial assimilation process of N in rice roots. Ca supports the integrity of the membrane; thus, the lack of Ca^2+^ might affect the transport rates of N compounds.

Although the absorption rate of NH_4_^+^ from the culture solution (Figure 3A) was comparable to that of NO_3_^−^ (Figure 3B), the concentration of NH_4_^+^ in the xylem sap (Figure 2A) was much lower than that of NO_3_^−^ (Figure 2B). The low concentration of NH_4_^+^ may be related to the detoxification of ammonium. The sum of the concentrations of Asn and Gln was approximately equal to the NO_3_^−^ concentration in xylem sap in N treatment (Figure 4A). The result may imply that most of the absorbed NH_4_^+^ was transported in the form of Gln and Asn, while most of NO_3_^−^ is transported to the shoots via the xylem without being assimilated to amides in the rice roots [7,8,28]. In soybean plants cultivated with ammonium sulfate, Asn and Gln were the principal N forms in the xylem sap, but soybean plants grown with nitrate transported N mainly in the form of NO_3_^−^ [46]. The K treatment did not affect the Gln and Asn concentrations in the xylem sap different from NO_3_^−^. In the Mg (Figure 4D) and Ca (Figure 4E) treatments, the lack of Mg^2+^ (×0 Mg) and Ca^2+^ (×0 Ca) caused a significant decrease in the concentrations of Gln and Asn in the xylem sap as well as NO_3_^−^.

Although the concentrations of Glu and Asp were low, but these amino acids presented consistently without being affected by the concentration treatments of N, P, K, Mg, and Ca (Figure 4). Glu and Asp were also the components of phloem sap in rice [27,28], therefore, Glu and Asn may be recycled in the xylem and phloem transport systems at a constant rate.

In the P treatment (Figure 4B), the concentrations of most N compounds were not significantly different among the P concentrations. This result might be due to the sufficient P storage in the roots and other organs, ensuring the P supply to support N metabolism. When the nodulated soybean plants were grown in P-deficient conditions, the Pi concentrations in the xylem sap decreased gradually for 15 days, but Pi was not depleted [47].

### 3.4. Effects of Major Nutrient Concentrations on Cation-Anion Balance and Organic Acids Accumulation in Xylem Sap

It is well known that the concentrations of equivalents of cations and anions in xylem sap should be almost the same to maintain a neutral electric balance. Van Beusichem et al. [48] reported the cationic and anionic composition of xylem exudates collected from castor oil plants supplied with NO_3_^−^ or NH_4_^+^ in a culture solution. With either N nutrition form, the total cation (K, Na, Ca, Mg, and NH_4_) concentration (mEq/L) in the xylem sap was almost equal to the total anion concentration (organic acids, P, Cl, NO_3_, plus SO_4_). They also reported that a high concentration of citrate and malate was detected in the xylem sap of plants cultivated with NH_4_^+^.

In this experiment, the patterns of the total equivalent concentrations of anions and cations in the xylem sap, shown in Figure 7, were similar for the anions and cations in each treatment. However, the total equivalent concentrations of anions tended to be lower than those of cations in the N, P, K, and Mg treatments, suggesting that some anions that could not be analyzed might compensate for lower anion concentrations.

The amides Gln and Asn have electric charges of 0 at pH 5.6–6.0 [49]; therefore, these compounds did not contribute to maintaining the cation-anion balance. Acidic amino acids such as Glu and Asp give an electric charge of −1 [50]; thus, they may contribute to compensating for the anion decrease. The Glu and Asp were always present in the xylem sap, but the concentrations of them were low and did not change by concentration treatment of N, P, K, Mg, and Ca. Some organic acids other than Cit and Mal were not analyzed in this study and might have been utilized by the anions to maintain the cation-anion balance in the xylem sap.

In this research, the Cit and Mal were remarkably accumulated in the xylem sap in the ×0 N treatment compared with the control (×1 N) (Figure 7A), which might be induced to compensate for the electric imbalance caused by the decrease in NO_3_^−^ in the xylem sap [51]. Van Beusichem et al. proposed that excess cation uptake is associated with a corresponding net release of H^+^ into the medium and, thus, an increase in cellular OH^−^, which induces the accumulation of organic acids, especially malate [48].

In the ×0 Mg treatment (Figure 7H), the Ca^2+^ concentration in xylem sap increased. However, the Mg^2+^ concentration did not increase in the ×0 Ca treatment (Figure 7J). Both Mg^2+^ and Ca^2+^ are divalent cations; the compensation mechanisms for equalizing the electric balance might be different.

### 3.5. The Applicability of Xylem Sap Analysis for the Diagnosis of Nutritional Status in Rice Plants

In this report, the concentrations of N, P, K, Mg, and Ca in the xylem sap responded to the concentrations of these elements after 3 days of the concentration treatments (Figure 2). In addition, the concentrations of these elements in xylem sap appeared to resemble the absorption rates of these elements (Figure 3). The result may imply the applicability of xylem sap concentrations of N forms, Pi, K, Mg, or Ca on the diagnosis of the nutrient status of the rice plants grown in the field. Practically, the timing and the amount of the application of the top dressing of N fertilizer are crucial for rice production by judging the N status in rice plants. In the diagnosis of N nutrition, leaf color or SPAD values are used to judge the N status of rice. The analysis of Gln, Asn, and the other elements in the xylem sap is beneficial to monitoring the plant nutrient status and soil availability of nutrients.

## 4. Materials and Methods

### 4.1. Plant Cultivation

Rice (*Oryza sativa* [L.], cv. Nipponbare) seeds were germinated on a wet paper towel for 5–6 days until the shoot length reached 1–2 cm. Then, about 25 germinated seedlings were transplanted for nursery cultivation into a 5 L pot with half-strength modified Kimura B solution (Table 1) for the next 5–6 days until the shoot length became 10–15 cm [52,53,54]. The pH of the culture solution was adjusted to 6.0 ± 0.2 using either 0.1 M HCl or 0.1 M NaOH. Half-strength Kimura B solution is popular for growing rice seedlings because rice plants have not shown deficiency or excess symptoms grown with this formula. The 8 rice seedlings with 10–15 cm shoots were transplanted to the second nursery pot with 5 L of 1/2 Kimura B solution and cultivated for the following 1 week. Then, each plant was transplanted in a 900 mL glass bottle with 850 mL of culture solution covered with aluminum foil to shade the culture solution. The culture solution was changed every 2 or 3 days. The plants were cultivated in a biophotochamber (LH-350S; Nippon Medical & Chemical Instruments Co. Ltd., Osaka, Japan) under 28 °C day/24 °C night temperatures, with 55% relative humidity. The photon flux density was 228 μmol m^−2^ s^−1^ with a 14-h photoperiod from 6 a.m. to 8 p.m. and a 10-h dark period from 8 p.m. to 6 a.m. The experiment was conducted with four biological replications. The plants were cultivated using a random arrangement in a growth chamber.

### 4.2. N, P, K, Mg, and Ca Treatments

The N, P, K, Mg, and Ca treatments were conducted for 3 days at 28–30 days after planting (DAP). For each treatment, five levels of culture solution, 0, 0.2, 0.5, 1, or 5 times the standard solution, were supplied. For the N treatment (Table 2A), the standard solution contained 370 μM Ca(NO_3_)_2_, 180 μM KNO_3_, and 370 μM (NH_4_)_2_SO_4_. The 0, 0.2, and 0.5 times solutions contained 0, 74, or 185 μM Ca(NO_3_)_2_; 0, 36, or 90 μM KNO_3_; and 0, 74, or 185 μM (NH_4_)_2_SO_4_, respectively, and the reduced Ca and K were replaced by CaCl_2_ and KCl to keep Ca and K concentration constant. The concentration of 5 times the standard solution was prepared simply by adding 1850 μM Ca(NO_3_)_2_, 900 μM KNO_3_, and 1850 μM (NH_4_)_2_SO_4_. In the P treatment (Table 2B), the KH_2_PO_4_ concentration was changed and the reduced K was supplied in the form of KCl. For the K treatment (Table 2C), the KNO_3_ and KH_2_PO_4_ concentrations were changed and replaced with NH_4_NO_3_ and NH_4_H_2_PO_4_. The increase in the NH_4_ concentration was adjusted by reducing the (NH_4_)_2_SO_4_ concentration. The concentrations of 0, 0.2, 0.5, 1, and 5 times Mg in the culture solution for the Mg treatment were prepared by changing the MgSO_4_ concentration to 0, 110, 275, 550, and 2750 μM, respectively (Table 2D). In the Ca treatment (Table 2E), the concentrations of 0, 0.2, 0.5, 1, and 5 times Ca in the culture solution were prepared by changing the Ca(NO_3_)_2_ concentrations to 0, 74, 185, 370, and 1850 μM, respectively. The lowered concentrations of Ca(NO_3_)_2_ were replaced with Mg(NO_3_)_2_ to keep NO_3_^−^ concentration constant among concentrations, and the concentrations of MgSO_4_ were adjusted to maintain Mg concentrations constant.

During the treatment period of 3 days, the culture solutions changed every day at about 10 AM. The transpiration rate was determined by weighing the culture solution in a glass bottle every day before and after cultivation. 1 mL of the culture solutions before and after cultivation were put in 1.5 mL plastic tubes and stored at −20 °C until analysis. The daily absorption rate of each nutrient element was determined by subtracting the content of the elements in the culture solution after one day of cultivation from that in the initial solution before cultivation. The absorption rate (μM/h) was calculated by dividing the daily absorption rate by 24. The absorption rates were determined based on the DW in grams of the roots (μM/g DW/h).

**Figure 10:**
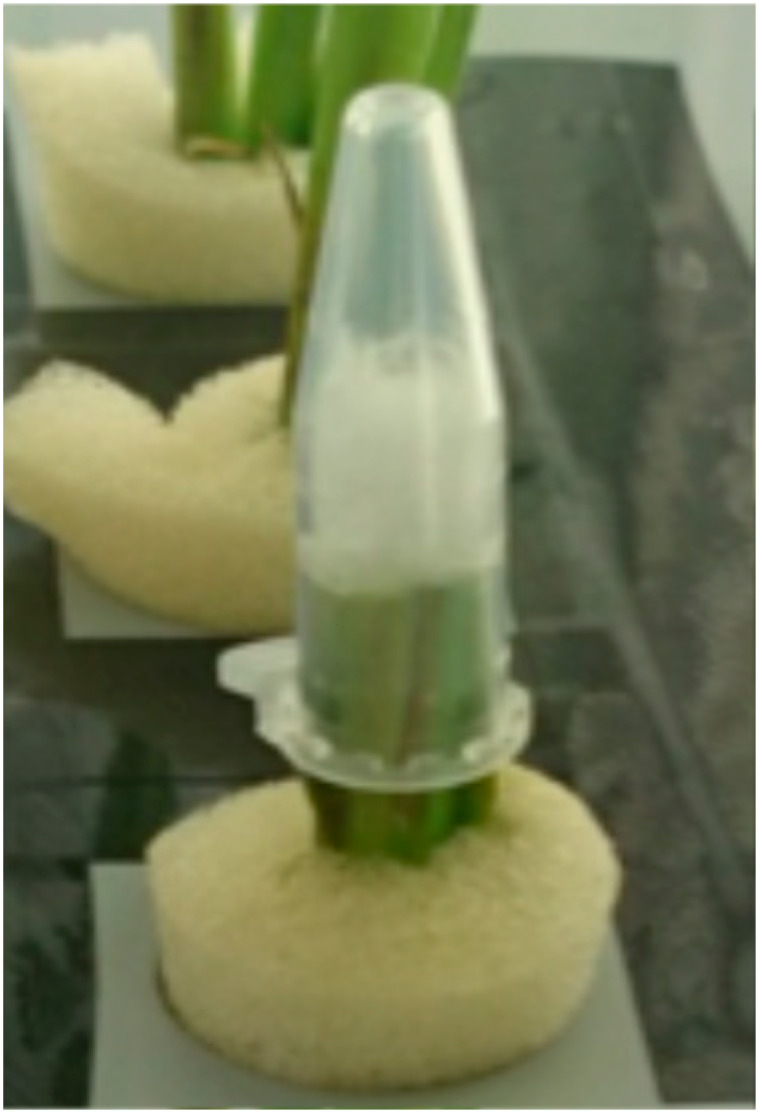
Xylem sap collection from rice stamp. The basal part of the shoot was cut by a razor blade, and exudating xylem sap was collected in quartz wool in a plastic tube for 1 h.

The collection of xylem sap from the cut shoot was done by a similar method used in soybean plants [46,47]. The first rice shoot was decapitated at about 3 cm above the node using a razor blade, and the xylem sap exudated from the decapitated stump was collected in quartz wool in a plastic tube for a period of 1 h from 9 a.m. to 10 a.m. (Figure 10). Then, the xylem saps of the second plants were collected for 1 h from 9.03 to 10.03 a.m., and the xylem saps of the following plants were collected with 3 min intervals. The xylem sap exudation rate (mL/h) was determined by weighing the plastic tube before and after collecting the xylem sap. The xylem sap was diluted with distilled water, filled up to 1 mL, and then stored at −20 °C until analysis. The roots were washed with distilled water, and the shoot and roots of the plants were dried in a ventilation oven at 70 °C for a few days, reaching the constant weight.

### 4.3. Analyses of Concentrations of Mineral Nutrients and N Compounds in Xylem Sap and Culture Solution

The concentrations of chloride, nitrate, sulfate, phosphate, Gln, Asn, Glu, Asp, Mal, and Cit in the xylem sap were analyzed via capillary electrophoresis (7100, Agilent Technologies, Inc., Santa Clara, CA, USA) using a fused silica tube (inner diameter: 50 μm; length: 104 cm) and a commercial buffer solution (α-AFQ109, Ohtsuka Electronics Co., Ltd., Osaka, Japan), with an applied voltage of 25 kV. The signals of each component were detected with a signal wavelength of 400 nm and a reference wavelength of 265 nm [55]. The concentrations of inorganic anions and cations in the xylem sap and culture solution were determined using ion chromatography (IC-2010, Tosoh Techno System, Inc., Tokyo, Japan) with a cation column (TSKgel superIC-Anion) or an anion column (TSKgel superIC-Cation). To compare the anion-cation balance in the xylem sap, the chemical equivalents were calculated based on the pH of the xylem sap, which was about 5.8 [56]. The occurrence of ionic species of Pi depends on the pH of the solution; H_2_PO_4_^−^ is a primary Pi species at pH 5.8 [57], with a valency of −1. Similarly, at pH 5.8, the average valencies of Cit [58] and Mal [59] are −2 and −1.67, respectively.

### 4.4. Statistics

Statistical significance was determined based on Tukey’s test using the statistical analysis program of Biomedical Statistics, Graduate School of Medicine, Osaka University [60].

## 5. Conclusions

In this study, the effects of varying N, P, K, Mg, and Ca concentrations in culture solution on nitrogen transport in the xylem sap of rice plants were investigated to evaluate the relationship between the absorption and transport rates of the major elements and the concentrations of N transport forms in xylem sap. The rice plants were treated with five concentrations of N, P, K, Mg, and Ca at 0, 0.2, 0.5, 1, and 5 times a control solution for three days. After the treatment, the concentrations of major N compounds, nitrate, ammonium, glutamine, asparagine, glutamate, and aspartate in the xylem sap were determined. In the control plants grown with the standard solution, nitrate, glutamine, and asparagine were the principal N compounds identified in the xylem sap. The concentrations of these N compounds decreased consistently with a decrease in the N concentration in the solution. High concentrations of organic acids, citrate, and malate in the xylem sap were observed under N-deficient conditions. This result is possibly due to the compensation of the cation-anion balance in the xylem sap. Different P concentrations did not affect the concentrations of N components in the xylem sap. With a decrease in the K concentration, only the nitrate concentration decreased, which was possibly due to the cation-anion balance being maintained. The glutamine and asparagine concentrations were decreased with a decrease in the Mg concentration. The Ca concentration did not affect the N concentration, except for under Ca deprivation conditions. The patterns in the concentrations of N, P, K, and Mg in the xylem sap collected from rice were similar to those in the absorption rates of these elements. Therefore, analyses of xylem sap can be used for nutritional diagnoses of rice plants to support precise fertilizer management.

## Figures and Tables

**Table 1:** Composition of the culture solution.

Nutrient Element	Chemical Formula	Concentration (μM)
N, Ca	Ca(NO_3_)_2_	370
N, K	KNO_3_	180
N, S	(NH_4_)_2_SO_4_	370
Mg, S	MgSO_4_	550
K, P	KH_2_PO_4_	210
Mn, Cl	MnCl_2_	7.3
B	H_3_BO_3_	9.3
Zn	ZnSO_4_	0.15
Cu	CuSO_4_	0.16
Mo	(NH_4_)_6_Mo_7_O_24_	0.015
Fe, Na	C_10_H_12_FeN_2_NaO_8_ 3H_2_O	90

The pH of the culture solution was adjusted to 6.0 ± 0.2 using either 0.1 M HCl or 0.1 M NaOH.

**Table 2:** (**A**) Concentrations in the culture solution in the N treatment (μM) ^1^. (**B**) Concentrations in the culture solution in the P treatment (μM) ^1^. (**C**) Concentrations in the culture solution in the K treatment (μM) ^1^. (**D**) Concentrations in the culture solution in the Mg treatment (μM) ^1^. (**E**) Concentrations in the culture solution in the Ca treatment (μM) ^1^.

(**A**)					
	**×0 N**	**×0.2 N**	**×0.5 N**	**×1 N**	**×5 N**
Ca(NO_3_)_2_	0	74	185	370	1850
KNO_3_	0	36	90	180	900
(NH_4_)_2_SO_4_	0	74	185	370	1850
CaCl_2_	370	296	185	0	0
KCl	180	144	90	0	0
(**B**)					
	**×0 P**	**×0.2 P**	**×0.5 P**	**×1 P**	**×5 P**
KH_2_PO_4_	0	42	105	210	1050
KCl	210	168	105	0	0
(**C**)					
	**×0 K**	**×0.2 K**	**×0.5 K**	**×1 K**	**×5 K**
KNO_3_	0	36	90	180	900
KH_2_PO_4_	0	42	105	210	1050
NH_4_NO_3_	180	144	90	0	0
NH_4_H_2_PO_4_	210	168	105	0	0
(NH_4_)_2_SO_4_	175	214	272.5	370	370
(**D**)					
	**×0 Mg**	**×0.2 Mg**	**×0.5 Mg**	**×1 Mg**	**×5 Mg**
MgSO_4_	0	110	275	550	2750
(**E**)					
	**×0 Ca**	**×0.2 Ca**	**×0.5 Ca**	**×1 Ca**	**×5 Ca**
Ca(NO_3_)_2_	0	74	185	370	1850
Mg(NO_3_)_2_	370	296	185	0	0
MgSO_4_	180	254	365	550	550

^1^ Concentrations of other nutrients are the same as in Table 1.

## Data Availability

The raw data supporting the conclusions of this article will be made available by the authors upon request.
